# Neurofibrillary tangle-predominant dementia followed by amyloid β pathology: a clinico-radio-pathological case providing insights into current disease-modifying therapeutic strategy

**DOI:** 10.1186/s40478-024-01815-x

**Published:** 2024-06-17

**Authors:** Tomoyasu Matsubara, Kenji Ishii, Yoko Saito, Aya Midori Tokumaru, Akira Arakawa, Manato Hara, Masanori Kurihara, Renpei Sengoku, Kazutomi Kanemaru, Atsushi Iwata, Tomio Arai, Akinori Miyashita, Takeshi Ikeuchi, Masato Hasegawa, Shigeo Murayama, Yuko Saito

**Affiliations:** 1 Department of Neuropathology (the Brain Bank for Aging Research [BBAR]), Tokyo Metropolitan Institute for Geriatrics and Gerontology, 35-2 Sakae-cho, Itabashi-ku, Tokyo, 173-0015 Japan; 2 Research Team for Neuroimaging, Tokyo Metropolitan Institute for Geriatrics and Gerontology, Tokyo, Japan; 3 Department of Neurology, Tokyo Metropolitan Institute for Geriatrics and Gerontology, Tokyo, Japan; 4 Department of Diagnostic Radiology, Tokyo Metropolitan Institute for Geriatrics and Gerontology, Tokyo, Japan; 5 Department of Neurology, The Jikei University Daisan Hospital, Tokyo, Japan; 6 Department of Pathology, Tokyo Metropolitan Institute for Geriatrics and Gerontology, Tokyo, Japan; 7 Department of Molecular Genetics, Brain Research Institute, Niigata University, Niigata, Japan; 8 Department of Brain and Neurosciences, Tokyo Metropolitan Institute of Medical Science, Tokyo, Japan; 9 Brain Bank for Neurodevelopmental, Neurological and Psychiatric Disorders, United Graduate School of Child Development, Osaka University, Osaka, Japan

Neurofibrillary tangle (NFT)-predominant dementia/primary age-related tauopathy (NFTD/PART) is an entity presenting with the deposition of NFT mainly in the medial temporal lobe. It is distinct from Alzheimer’s disease (AD) in that it shows minimal deposition of amyloid β (Aβ) plaques [3, 6, 10]. Recently, based on the amyloid cascade hypothesis, Aβ-targeting disease-modifying therapies (DMTs) for AD have been developed [9]. The dilemma, however, is that Aβ deposition is observed frequently in the advanced ages regardless of the presence of AD [8]. Thus, Aβ plaque accumulation can occur concurrently in patients presenting with other dementing conditions. This situation complicates the efficacy of DMTs, making accurate differentiation between conditions crucial for effective treatment, especially considering the high costs and potential side effects of DMTs. Here, we report a clinico-radio-pathologically-confirmed case that initially developed NFTD/PART and then gradually developed overlapping Aβ pathology, highlighting its importance in the context of DMTs.

An 88-year-old man without a significant past medical history or family history of neurological disorders presented with mild cognitive decline (Mini-Mental State Examination [MMSE] score: 26/30). Bilateral atrophy and reduced radioligand uptake in the medial temporal lobes were observed on MRI and [^18^F]-2-fluoro-2-deoxy-D-glucose positron emission tomography (FDG-PET), respectively (Fig. 1). At age 90, cerebrospinal fluid Aβ, phosphorylated tau, and tau levels were normal. [^11^C]-Pittsburgh compound-B (PiB) PET, detecting both diffuse and cored Aβ plaques [5], also showed no significant Aβ deposition. At age 91, he presented with topographical disorientation and showed a lower MMSE score (total 20/30; word recall 0/3). Based on these findings, the patient received a diagnosis of non-Aβ-related dementia, such as argyrophilic grain dementia or NFTD/PART. Subsequently, a cognitive impairment progressed slowly. Subsequent PiB PET at age 92 revealed focal Aβ deposition in the parietal and temporal lobes and at age 97 Aβ deposition throughout the cerebrum (Fig. 1a). In contrast, the overall brain atrophy progressed only mildly despite the 9-year follow-up interval under the dementing condition, while the hippocampal atrophy became more pronounced, extending to the posterior regions (Fig. 1b). The patient died of aspiration pneumonia at age 97.

**Fig. 1:**
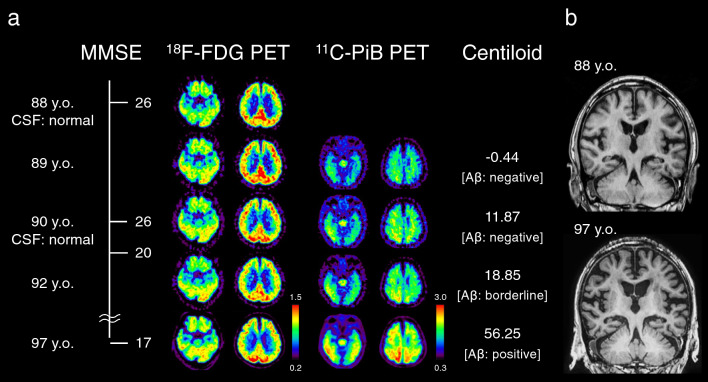
Chronological clinical and positron emission tomography (PET) findings (**a**). Presenting with mild cognitive impairment, bilaterally reduced radioligand uptake in the medial temporal lobes was observed on [18F]-2-fluoro-2-deoxy-D-glucose (FDG)-PET. However, cerebrospinal fluid (CSF) amyloid β (Aβ), phosphorylated tau, and tau levels were normal, and [11C]-Pittsburgh compound-B (PiB) PET also indicated no significant Aβ deposition. After that, with the progression of cognitive dysfunction, there was a gradual increase in Aβ accumulation in the cerebrum. Centiloid scaling was used to standardize the results of amyloid PET quantitatively. Centiloid scale values range from 0 to 100, with 0 representing definitively Aβ-negative brains of young, healthy controls and 100 representing the average signal observed in patients with mild to moderate dementia due to Alzheimer’s disease. Centiloid scale transition also supports the fact that Aβ accumulation began after the onset of dementia and showed a slow increase. Color bars indicate the standardized uptake value ratio (SUVR). Comparison of T1-weighted MRI images at ages 88 and 97 (**b**). The overall brain atrophy progressed only modestly despite the 9-year follow-up interval under the dementing condition, while the hippocampal atrophy became more pronounced, extending to the posterior regions. Abbreviations: *MMSE* Mini-Mental State Examination score, *y.o.* years old

Autopsy was performed 5 h after death. The brain weighed 1,137 g before fixation. A detailed description of the neuropathological examination methods is provided in Supplementary Information. Macroscopic examination revealed bilateral hippocampal atrophy (Fig. 2a). Microscopic examination revealed severe neuronal loss (Fig. 2b) and gliosis in the hippocampal CA1 region with abundant NFTs (Fig. 2c, d), predominantly extracellular ghost tangles (Fig. 2e, f). Many NFTs were observed in the hippocampus, including the dentate gyrus. NFTs were confined to the temporal lobe (Fig. 2g), while there was minimal distribution in other neocortices (Braak NFT stage IV). These NFTs were immunoreactive for both 4-repeat and 3-repeat tau. Aβ plaques were present throughout the cortex and striatum; however, their amount was up to moderate, and the plaques were mainly diffuse plaques as well as cored plaques (corresponding to Thal phase: 3, CERAD score: moderate) (Fig. 2h). Other aging/dementia-related pathologies were as follows: BBAR Lewy stage II (prodromal stage), Saito argyrophilic grain stage I, no TDP-43 pathology was identified. The vascular pathology was also minimal.

**Fig. 2:**
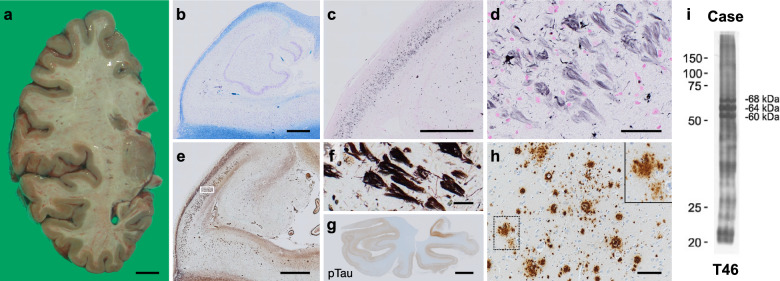
Neuropathological findings. Macroscopic examination showed selective hippocampal atrophy (**a**). Klüver–Barrera staining revealed severe neuronal loss in the hippocampal CA1 region (**b**). Gallyas staining showed abundant neurofibrillary tangles (NFTs), predominantly extracellular ghost tangles (**c**, **d**). Methenamine silver staining showed few plaques but abundant extracellular ghost tangles (**e**, **f** [magnified image of square in panel **e**]). Phosphorylated tau immunostaining showed NFTs distributed in the temporal lobe (**g**). Amyloid β immunostaining shows a mixture of diffuse and cored plaques in amounts up to moderate (**h**; taken in the middle temporal gyrus). Immunoblot analysis of sarkosyl-insoluble tau. Western blotting with an anti-tau antibody (clone T46, mouse monoclonal, dilution 1:1000, Thermo Fisher Scientific, Rockford, USA) detected full-length hyperphosphorylated tau bands with apparent molecular weight of 60, 64, and 68 kDa, consistent with NFT-predominant dementia/primary age-related tauopathy or Alzheimer’s disease tau banding pattern (**i**). Scale bars: 1 cm (**a**), 1 mm (**b**, **c**, and **e**), 50 μm (**d**), 20 μm (**f**), 5 mm (**g**), and 100 μm (**h**)

In addition, immunoblotting of the temporal cortex tissue revealed phosphorylated triplet tau bands (60, 64, and 68 kDa), consistent with NFTD/PART or AD. The patient’s *APOE* genotype was ε3/3. No *MAPT* pathological mutation was detected.

Clinico-radiologically, the patient exhibited cognitive decline preceding sufficient Aβ deposition, and, during the disease course, Aβ deposition became progressively evident. Neuropathological analysis displayed a characteristic finding of NFTs densely filling the hippocampus, consistent with advanced NFTD/PART [10]. The amount of Aβ plaques was also relatively small, and diffuse plaques predominated, which is not typical for AD. Although the brain condition at autopsy was categorized as intermediate AD according to the National Institute on Aging-Alzheimer’s Association criteria [7] and did not meet the NFTD/PART criteria [3, 10], the longitudinal PET findings suggest that the pathological findings would have met the criteria for NFTD/PART if the brain was examined at a little earlier stage in his life. Namely, the longitudinal clinicoradiological observations combined with the autopsy results indicate that NFTD/PART pathology was initially developed, followed by a subsequent overlap with Aβ pathology. In fact, this patient’s slower progression of cognitive decline, which deviated from the expected annual MMSE score decrement associated with AD (approximately 3 points/year) [2, 4], is consistent with the characteristics of NFTD/PART [1]. Furthermore, the mean age at onset of NFTD/PART has been documented as 89 years [range 81–95 years] [11]. Indeed, this patient also developed dementia at around 90 years of age. Therefore, NFTD/PART should be considered in the differential diagnosis of patients with the features described above; in addition, it should be noted that NFTD/PART may sometimes be comorbid with mild Aβ pathology with aging.

Recognizing the existence of such overlapping pathological conditions is becoming increasingly important with the advent of Aβ-targeting DMTs for AD. In such cases where sufficient tau accumulation for the dementing condition development precedes sufficient Aβ deposition, “tau-first condition”, tau accumulation and neuronal loss may be substantially advanced relative to Aβ levels. This condition may lead to a misidentification of the appropriate treatment subjects, potentially reducing the efficacy of Aβ-targeting DMTs in delaying the dementia progression compared with the AD cases obediently following the amyloid cascade hypothesis, “amyloid-first condition”. Thus, the existence of such overlapping conditions underscores the need to deeply understand the heterogeneity of the current DMT candidates and optimize the DMT eligibles.

In conclusion, this NFTD/PART case with overlapping Aβ pathology illustrates the complexity of diagnosing and treating neurodegenerative diseases, particularly in an aging population. This highlights the need for a more comprehensive understanding of the pathological basis of dementia syndromes which extends beyond the simplistic Aβ-centric model of AD.

### Supplementary Information

Supplementary Material 1.

## Data Availability

The datasets used and/or analyzed during the current study are available from the corresponding author upon reasonable request.

## Abbreviations

AD: Alzheimer’s disease
Aβ: Amyloid β
BBAR: The Brain Bank for Aging Research
DMT: Disease-modifying therapy
FDG: [^18^F]-2-fluoro-2-deoxy-D-glucose
MMSE: Mini-Mental State Examination
NFT: Neurofibrillary tangle
NFTD/PART: NFT-predominant dementia/primary age-related tauopathy
PET: Positron emission tomography
PiB: [^11^C]-Pittsburgh compound-B
