# Supplementary material for: Neurofibrillary tangle-predominant dementia followed by amyloid β pathology: a clinico-radio-pathological case providing insights into current disease-modifying therapeutic strategy
Source: Acta Neuropathol Commun. 2024 Jun 17;12:98. doi: 10.1186/s40478-024-01815-x (PMC11181578; doi:10.1186/s40478-024-01815-x)
Supplement: Supplementary file 1 — Supplementary Material 1. [file 40478_2024_1815_MOESM1_ESM.docx]

**Supplementary Information**

**Neuropathological examination**

The left half of the brain was fixed in 20% buffered formalin, and the right half was sliced and frozen. The cerebral hemisphere, brainstem, and cerebellum were dissected in the coronal, axial, and sagittal planes, respectively. Representative anatomical areas were embedded in paraffin, and 6-μm-thick sections were stained with hematoxylin and eosin, Klüver–Barrera, and by Gallyas–Braak and methenamine silver impregnation. Subsequently, immunoreaction product deposits on immunohistochemically stained sections were visualized with Ventana BenchMark GX autostainer (Ventana Medical Systems, Tucson, AZ, USA), I-View Universal DAB Detection Kit (Roche, Basel, Switzerland), and primary antibodies against, phosphorylated tau (AT8; mouse-monoclonal, 1:1,000, Innogenetics, Ghent, Belgium), amyloid β (12B2; mouse-monoclonal, 1:50 with formic acid for antigen retrieval, IBL, Gunma, Japan), phosphorylated α-synuclein (pSyn#64; mouse-monoclonal, 1:20,000 with formic acid for antigen retrieval, FUJIFILM Wako Pure Chemical Corporation, Osaka, Japan), phosphorylated TDP43 (11-9, mouse-monoclonal, 1:10,000 with microwave in Dako target retrieval solution [pH 6.0] for antigen retrieval, Cosmo Bio, Tokyo, Japan), 4-repeat tau (1E1/A6; mouse-monoclonal, 1:50 with microwave and formic acid, Upstate, Lake Placid, NY, USA), and 3-repeat tau (8E6/C11, mouse-monoclonal, 1:2000 with microwave in Dako target retrieval solution [pH 6.0] and formic acid, Upstate, Lake Placid, NY, USA). The specimen was investigated with a light microscope (Eclipse Ni, Nikon, Tokyo, Japan) and photographed using a digital camera (DS-Ri2, Nikon, Tokyo, Japan). Neuropathological grading was performed according to the following criteria: Braak NFT stage [1], Thal amyloid phase [5], CERAD neuritic plaque score [2], BBAR Lewy stage [4], Saito argyrophilic grain stage [3].

**References**

1. Braak H, Braak E (1991) Neuropathological stageing of Alzheimer-related changes. Acta Neuropathol 82:239–59. https://doi.org/10.1007/BF00308809

2. Mirra SS, Heyman A, McKeel D, Sumi SM, Crain BJ, Brownlee LM, et al (1991) The Consortium to Establish a Registry for Alzheimer's Disease (CERAD). Part II. Standardization of the neuropathologic assessment of Alzheimer's disease. Neurology 41:479-86. https://doi.org/10.1212/wnl.41.4.479

3. Saito Y, Ruberu NN, Sawabe M, Arai T, Tanaka N, Kakuta Y, et al (2004) Staging of argyrophilic grains: an age-associated tauopathy. J Neuropathol Exp Neurol 63:911–8. https://doi.org/10.1093/jnen/63.9.911

4. Tanei ZI, Saito Y, Ito S, Matsubara T, Motoda A, Yamazaki M, et al (2021) Lewy pathology of the esophagus correlates with the progression of Lewy body disease: a Japanese cohort study of autopsy cases. Acta Neuropathol 141:25–37. https://doi.org/10.1007/s00401-020-02233-8

5. Thal DR, Rüb U, Orantes M, Braak H (2002) Phases of A beta-deposition in the human brain and its relevance for the development of AD. Neurology 58:1791-800. https://doi.org/10.1212/wnl.58.12.1791
